# Adult vaccination in people living with HIV attending a tertiary centre infectious diseases clinic

**DOI:** 10.4102/sajhivmed.v27i1.1819

**Published:** 2026-09-25

**Authors:** Mitchell T. Muchichwa, Lyle W. Murray, Sarah Stacey, Charles Feldman

**Affiliations:** 1 School of Pathology, Faculty of Health Sciences, University of the Witwatersrand, Johannesburg, South Africa; 2 Division of Infectious Diseases, Department of Internal Medicine, Faculty of Health Sciences, University of the Witwatersrand, Johannesburg, South Africa; 3 Department of Internal Medicine, Faculty of Health Sciences, University of the Witwatersrand, Johannesburg, South Africa

**Keywords:** vaccination, HIV, vaccine-preventable, knowledge, attitudes, acceptance, awareness, uptake, guidelines, hesitance

## Abstract

**Background:**

People living with HIV (PLWH) are at increased risk of acquiring vaccine-preventable diseases and of developing severe illness because of their underlying immunodeficiency. Despite this risk, vaccination coverage remains low among these patients. International and local guidelines exist to address immunisation recommendations in PLWH, but evidence on adherence, knowledge, and attitudes toward vaccination in this population remain limited.

**Objectives:**

The aim of the study was to investigate vaccination uptake, knowledge, and acceptance among PLWH.

**Method:**

Interviews and retrospective file reviews were undertaken among PLWH attending the Charlotte Maxeke Johannesburg Academic Hospital Infectious Diseases Clinic from 01 June 2023 to 30 June 2023.

**Results:**

A total of 107 PLWH were interviewed, of whom 72 were women (67.3%), with a mean age of 47 years. Most (76.6%) had a prior history of tuberculosis. Overall, 24.3% had received at least one vaccine after their HIV diagnosis, of which influenza was the most common (76.9%). Only four vaccines, namely those against influenza, hepatitis B, herpes zoster and pneumococcus, were known to, or had been received by, the patients. While 65% believed vaccines could prevent disease, and 41.1% thought they could be harmful, notably 67.2% were willing to accept a vaccine if recommended by a healthcare provider.

**Conclusion:**

Our study revealed low vaccination rates amongst PLWH in Johannesburg, South Africa, specifically regarding guidelines from the South African HIV Clinicians Society. Increased awareness of the influenza vaccine and direct recommendations from healthcare providers were key factors linked to vaccine uptake.

**What this study adds:** This study addresses the critical data gap regarding vaccine uptake among South African PLWH. It reveals low uptake (24.3%) and limited vaccine literacy. Crucially, it identifies healthcare provider recommendations as the primary catalyst for acceptance, providing a clear evidence-based strategy to improve clinical outcomes and guideline implementation.

## Introduction

Vaccine-preventable diseases (VPDs) continue to impose a significant clinical and economic burden in South Africa (SA) and globally. In the United States (US), an estimated 40 000 to 50 000 adults die from VPDs annually,^1^ with the burden disproportionately affecting at-risk populations, such as individuals at risk of severe clinical outcomes because of underlying comorbidities as well as those with a greater risk of exposure to VPDs.^2^ Countries in sub-Saharan Africa account for 34% of the global VPD burden,^3^ while at the same time bearing one of the world’s highest HIV burdens – an estimated 7.8 million people were living with HIV (PLWH) in South Africa in 2023.^4^

Hepatitis B virus (HBV) is one of the most common VPDs among PLWH because of overlapping transmission routes.^5,6^ In SA, HBV prevalence in this group ranges from 0.4% to 23%.^7^ Furthermore, despite antiretroviral therapy (ART), PLWH remain at heightened risk of invasive pneumococcal disease (IPD). Meiring et al.^8^ reported a 43-fold higher incidence of IPD in PLWH receiving ART compared to their HIV-negative peers. People living with HIV have an increased risk of mortality from human papilloma virus (HPV)-associated malignancies than the general population. This increased risk is particularly significant in women.^9^ Interestingly, the association between HIV and susceptibility to meningococcal infections has been debated across different countries. Earlier African studies did not find a clear link between HIV and meningococcal infections.^10,11^ However, Cohen et al.^12^ reported an 11-fold higher risk among PLWH in SA, and further studies are necessary to quantify the morbidity and mortality associated with meningococcal disease among PLWH. In Africa, there are limited data on the burden of varicella zoster virus (VZV) among PLWH.^7^ Diphtheria, pertussis, and tetanus follow similar trends,^13^ although global surveillance gaps limit understanding of their true burden in PLWH.

In 2018, the South African HIV Clinicians Society (SAHCS) published guidelines for the vaccination of HIV-positive adolescents and adults in South Africa.^7^ So far, these remain the only guidelines published in the African region and include similar recommendations to those of some international guidelines.^14,15^ The SAHCS guidelines advise individuals to receive vaccinations for influenza, pneumococcal disease, meningococcal disease, HPV, tetanus, diphtheria, and pertussis. Additionally, vaccinations for hepatitis A and B are recommended, as well as for measles, mumps and rubella (MMR) for individuals with CD4 cell counts above 200 cells/uL.^7^ The SAHCS guidelines do not recommend the live-attenuated herpes zoster vaccine (Zostavax) for PLWH because of limited epidemiological data supporting its use in Africa.^7^ In contrast, some international guidelines do recommend Zostavax for PLWH, but only those with CD4 cell counts above 200 cells/uL. This recommendation is based on promising clinical trial data and expert opinion, but with limited evidence on long-term effectiveness.^14,15^ The recombinant herpes zoster vaccine (Shingrix) was not available in South Africa at the time the SAHCS guideline was published.

The reasons for low vaccination rates among PLWH reported in many countries are multifactorial. A study investigating self-reported vaccination coverage in Germany found that CD4 cell count had the strongest association with vaccine uptake, with CD4 cell count above 200 cells/uL being associated with higher vaccination coverage.^16^ These results were corroborated in a study conducted by Johnson et al.,^17^ which found that participants with CD4 cell counts less than 200 cells/uL were approximately 9-fold less likely to be up to date on all vaccinations compared to those with CD4 cell counts above 200 cells/uL. An African cohort study assessing HIV clinic visit adherence found that the reason ‘Feeling ill’ accounted for one of the top five reasons for missed clinic appointments, and CD4 count < 200 cells/uL was significantly associated with missing expected visits.^18^ This suggests that illness associated with advanced HIV disease may play a role in the ability to follow up with clinic appointments that is reflected in adherence assessments. The relationship between immunological status, clinic attendance and vaccine uptake remains insufficiently explored, however, and warrants further investigation in the South African context. Other barriers to uptake often include lack of information, forgetfulness, and poor provider communication. In a Belgian study, 89% of PLWH were unaware of vaccine recommendations, 38% lacked information on vaccination, and 6% did not see the need for vaccination.^19^ A Ugandan study assessing the uptake and acceptability of COVID-19 vaccines found that 90.5% of PLWH trusted vaccine information from healthcare providers more than any other source.^20^

Concerns over vaccine safety and side effects also persist. In the United Kingdom (UK), 23% of PLWH refused the influenza vaccine because of a fear of adverse effects.^21^ However, a multicentre study involving 30 000 PLWH in the US found no long-term negative impact of influenza vaccination on CD4 cell counts, viral loads, or disease progression. It was further revealed that some patients not receiving ART experienced slight increases in HIV viral loads and reductions in CD4 cell counts following influenza vaccination, and most often normalised 2 to 6 weeks after vaccination.^22^

The SAHCS guidelines note that vaccines are often not available at the points of care where PLWH receive treatment, while vaccine cost may serve as an additional barrier to access.^7^ This gap may reflect structural barriers within healthcare systems, as vaccination services are not consistently integrated into routine HIV care. Despite these insights, there remains a paucity of data regarding the accessibility of vaccines recommended by the SAHCS for PLWH. The aim of this study was to investigate vaccine uptake and the factors influencing knowledge and acceptance of vaccination among PLWH attending a tertiary centre infectious diseases clinic, to inform policy and guide interventions to improve vaccine uptake.

## Research methods and design

This study used a prospective cross-sectional audit and retrospective record review. Patient interviews provided self-reported data on demographics, vaccine uptake, knowledge, and acceptance, while review of the patient files was used to verify HIV monitoring results and medical history.

Data collection was carried out from 01 to 30 June 2023 at the Infectious Diseases Clinic at Charlotte Maxeke Johannesburg Academic Hospital (CMJAH), a tertiary teaching hospital in Johannesburg, Gauteng, South Africa’s most urbanised province.^23^ Gauteng has the fifth highest HIV prevalence nationally (17.6%), with the highest burden among adolescent girls and young women (35%).^24^ The CMJAH Infectious Diseases Clinic serves approximately 14 400 to 19 200 patients annually.^25^ Adults (≥ 18 years) living with HIV were approached during their waiting times for consultation or medication pickup. On average the Infectious Disease Clinic receives approximately 60–80 patients per day. A convenience sampling method was used, whereby patients present at the clinic during the study period were approached and were invited to participate. Prior to the interview, patients willing to participate were provided with an information sheet and consent form, which outlined the study’s purpose and time commitment. The interviews took place in a private consulting room, away from any queues, and lasted no longer than 10 min per participant.

### Data analysis

Data were analysed using STATA version 17 (StataCorp LLC, College Station, Texas, United States). Descriptive statistics were used to summarise the data. For continuous variables, means and standard deviations (s.d.) were reported, or medians and interquartile ranges, where appropriate. Categorical variables were summarised using frequencies and percentages.

Associations between independent variables and vaccine uptake were assessed using *t*-tests for continuous variables, and chi-square or Fisher’s exact tests for categorical variables. A significance level of 5% was applied (*P* < 0.05). Logistic regression was used to explore associations and estimate the likelihood of vaccine uptake. Odds ratios (OR) with 95% confidence intervals (CI) were reported. Variables with *P*-values below 0.05 were included in the multivariable logistic regression analysis.

### Ethical considerations

Approval to conduct the study was granted by the University of the Witwatersrand Human Research Ethics Committee (Medical), reference M221136. Site approval was obtained from the Head of Internal Medicine and the Chief Executive Officer of Charlotte Maxeke Johannesburg Academic Hospital. Written informed consent was obtained from participants in English, Zulu, or Tswana. Those unable or unwilling to consent were excluded. Confidentiality was ensured via secure, password-protected electronic storage, and locked storage of paper forms.

## Results

A total of 107 PLWH were interviewed. Most respondents were women (67.3%), and the mean age was 47 years. The majority of participants had completed up to secondary level education (72.9%), and more than half (54.2%) were unemployed. Most participants (57.0%) reported living with HIV for between 11 and 20 years (Table 1).

**TABLE 1:** Demographic characteristics of study participants.

Variables	Level	Total cohort (*N* = 107)			Vaccinated (*N* = 26)			Unvaccinated (*N* = 81)			*P*
		*n*	s.d.	%	*n*	s.d.	%	*n*	s.d.	%	
**Age (years)**		47	10.4	-	47	9.4	-	47	10.8	-	0.910
**Sex**	Female	72	-	67.3	23	-	88.5	49	-	60.5	**0.008**
	Male	35	-	32.7	3	-	11.5	32	-	39.5	-
**Highest level of education**	Primary	11	-	10.3	2	-	7.7	9	-	11.1	0.300
	Secondary	78	-	72.9	17	-	65.4	61	-	75.3	-
	Tertiary	18	-	16.8	7	-	26.9	11	-	13.6	-
**Employment status**	Employed	49	-	45.8	13	-	50.0	36	-	44.4	0.620
	Unemployed	58	-	54.2	13	-	50.0	45	-	55.6	-
**Children**	Yes	95	-	88.8	26	-	100.0	69	-	85.2	**0.040**
	No	12	-	11.2	0	-	0.0	12	-	14.8	-
**Live with children**	Yes	63	-	58.9	18	-	69.2	45	-	66.2	0.790
	No	31	-	29.0	8	-	30.8	23	-	33.8	-

Note: Vaccinated, having received at least one recommended vaccine after HIV diagnosis; s.d., standard deviation; all other values are %, percentage. *P*-values were obtained using Student’s *t*-test, Pearson *χ*^2^ or Fisher’s exact test. *P*-values ≤ 0.05 are shown in bold. The *n* values for some demographic variables do not total 107 because of missing data (i.e. participants opted to not provide a response for that specific characteristic).

Among the patients, the most common prior or current infection was tuberculosis (TB) (76.6%), followed by shingles (17.1%) and HBV (15.1%). Nearly all participants (99.1%) were on ART, but only 36.2% were aware of their specific ART regimen. Awareness of key clinical indicators was low, with only 29.0% aware of their HIV viral load and 11.2% aware of their CD4 cell count. Among participants with available data, 76.3% had an undetectable viral load (< 50 copies/mL). The clinical characteristics of the study participants are detailed in Table 2.

**TABLE 2:** Clinical characteristics of study participants.

Variables	Level	Total cohort (*N* = 107)		Vaccinated (*N* = 26)		Unvaccinated (*N* = 81)		*P*
		*n*	%	*n*	%	*n*	%	
**Time living with HIV (years)**	≤ 1	4	3.7	1	3.9	3	3.9	-
	2–10	27	25.2	4	15.4	23	29.5	-
	11–20	61	57.0	15	57.7	46	59.0	-
	21–35	12	11.2	6	23.1	6	7.7	-
**Knowledge of own viral load**	Yes	31	29.0	12	46.2	19	23.8	**0.029**
	No	75	70.9	14	53.9	61	76.3	-
**Knowledge of own CD4 cell count**	Yes	12	11.2	6	23.1	6	7.5	0.067
	No	94	87.8	20	76.9	74	92.5	-
**Last CD4 cell count (cells/uL)**	< 200	26	24.3	5	26.3	21	33.9	0.269
	200–500	26	24.3	9	47.4	17	27.4	-
	> 500	29	27.1	5	26.3	24	38.7	-
**Last viral load (copies/mL)**	Undetectable	74	69.2	14	60.9	60	81.1	**0.010**
	< 500	14	13.0	8	34.8	6	8.1	-
	> 500	9	8.4	1	4.4	8	10.8	-
**ART status**	Yes	106	99.1	26	100	80	98.8	1.000
	No	1	0.9	0	0.0	1	1.2	-
**Knowledge of ART**	Yes	38	36.2	15	57.7	23	29.1	**0.009**
	No	67	63.8	11	42.3	56	70.9	-
**History of opportunistic infections and cervical cancer**	TB	82	76.6	17	65.4	65	80.2	-
	Pneumonia	9	8.6	1	3.9	8	10.1	-
	PJP	5	4.8	1	3.9	4	5.1	-
	Shingles	18	17.1	3	11.5	15	19.0	-
	Meningitis	8	7.6	2	7.7	6	7.6	-
	Hepatitis B	16	15.1	3	11.5	13	16.3	-
	Influenza	7	6.7	2	7.7	5	6.3	-
	Cervical cancer	3	2.9	1	4.2	2	2.6	-
**Comorbidities**	Hypertension	31	29.0	9	34.6	22	28.6	-
	Diabetes	10	9.3	4	15.4	6	7.7	-
	Asthma	3	2.8	1	3.9	2	2.6	-
	COPD	3	2.8	1	3.9	2	2.6	-
	Heart failure	9	8.4	2	7.7	7	9.1	-
	Kidney disease	13	12.1	1	3.9	12	15.8	-
	Cancer	3	2.8	1	3.9	2	2.6	-

Note: Vaccinated, having received at least one recommended vaccine after HIV diagnosis; ART, antiretroviral therapy; COPD, chronic obstructive pulmonary disease; PJP, *Pneumocystis jirovecii* pneumonia; TB, tuberculosis. *P*-values were calculated using the Pearson’s *χ*^2^ and Fisher’s exact test. *P*-values ≤ 0.05 are shown in bold. Percentages are calculated from the total study sample; however, the *n* values for some demographic variables do not total 107 because of missing data. *P*-values were not calculated for variables with multiple categories or small cell sizes, as these did not meet the assumptions required for valid statistical testing and were therefore presented descriptively.

### Vaccination uptake, knowledge, and acceptance

Overall adult vaccination uptake was low, with only 24.3% of PLWH having received at least one vaccine, other than COVID-19, since their HIV diagnosis. Among the vaccinated group (*n* = 26), the most common vaccine received was influenza (76.9%), followed by hepatitis B (19.2%). Awareness of recommended vaccines for PLWH was limited – the influenza vaccine was the most commonly known (59.8%), while knowledge of all other recommended vaccines was below 20%.

Although 64.5% of participants believed vaccination would help prevent disease, a notable proportion (41.1%) thought vaccines could be harmful. Importantly, 67.3% were willing to accept a vaccine if recommended by a healthcare provider, but only 28.0% had ever been advised by a doctor or nurse to get vaccinated. The most frequently cited factors influencing the decision to vaccinate were knowledge of the vaccine (68.2%) and fear of side effects (50.5%). Knowledge, history, and acceptance data regarding vaccination are shown in Table 3.

**TABLE 3:** Knowledge, history, and acceptance of vaccination among the study cohort.

Question/Variable	Answer option	Total cohort (*N* = 107)		Vaccinated (*N* = 26)		Unvaccinated (*N* = 81)		*P*
		*n*	%	*n*	%	*n*	%	
**Awareness and receipt of vaccination**								
What childhood vaccinations are you aware of?	None	59	55.1	10	38.5	49	60.5	**0.050**
	≥ 1	45	42.0	15	57.7	30	37.0	-
Are you aware of having received any childhood vaccinations?	Yes	81	75.7	21	80.8	60	74.1	0.490
	No	26	24.3	5	19.2	21	25.9	-
Have you received any vaccine since being diagnosed with HIV (apart from COVID-19)	Yes	26	24.3	-	-	-	-	-
	No	81	75.7	-	-	-	-	-
Which adult vaccines have been received?	Influenza	20	18.70	-	-	-	-	-
	Hepatitis B	5	4.7	-	-	-	-	-
	Shingles	1	1.0	-	-	-	-	-
	Pneumococcal	1	1.0	-	-	-	-	-
Those aware of the following vaccines in adults	Pneumococcal	5	4.7	1	3.9	4	4.9	1.000
	Influenza	64	59.8	23	88.5	41	50.6	**0.001**
	Hepatitis B	15	14.0	6	23.1	9	11.1	0.190
	HPV	4	3.7	0	0.0	4	4.9	0.570
	MMR	18	16.8	0	0.0	18	22.2	**0.006**
	TDaP	6	5.6	0	0.0	6	7.4	0.330
	Shingles	18	16.8	4	15.4	14	17.2	1.000
**Attitudes and beliefs regarding vaccination**								
Do you think getting vaccinated is harmful to your body?	Yes	29	27.1	7	26.9	22	27.2	0.220
	No	44	41.1	14	53.9	30	37.0	-
	I don’t know	34	31.8	5	19.2	29	35.8	-
Do you think getting vaccinated against disease will help to prevent that disease?	Yes	69	64.5	16	61.5	53	66.3	0.880
	No	16	15.0	4	15.4	12	15.0	-
	I don’t know	21	19.6	6	23.1	15	18.8	-
Do you think that getting a vaccine could worsen your HIV?	Yes	18	16.8	2	7.7	16	19.8	0.300
	No	60	56.1	15	57.7	45	55.6	-
	I don’t know	29	27.1	9	34.6	20	24.7	-
**Receipt and acceptance of healthcare worker recommendations regarding vaccination**								
Have you ever been advised by your doctor or nurse to get vaccinated?	Yes	30	28.0	14	53.9	16	20.0	**0.001**
	No	76	71.0	12	46.2	64	80.0	-
If yes, which vaccines have been recommended to you?	Influenza	18	16.8	-	-	-	-	-
	Hepatitis B	6	5.6	-	-	-	-	-
	Shingles	4	3.7	-	-	-	-	-
	Pneumococcal	1	1.0	-	-	-	-	-
	COVID-19	5	4.7	-	-	-	-	-
If your doctor or nurse recommended a vaccine, would you accept it?	Yes	72	67.3	23	88.5	49	60.5	**0.031**
	No	17	15.9	2	7.7	15	18.5	-
	Not sure	18	16.8	1	3.9	17	20.9	-
Which factors would affect your decision to vaccinate?	Knowledge of vaccine	73	68.2	18	69.2	55	67.9	0.899
	Fear of side effects	54	50.5	9	34.6	45	55.6	0.063
	Availability of vaccine	21	19.6	7	26.9	14	17.3	0.282
	Family discussions	9	8.4	4	15.4	5	6.2	0.216
	Cost	30	28.0	8	30.8	22	27.2	0.722
	Mainstream media	28	26.2	9	34.6	19	23.5	0.260
	Social media	14	13.1	4	15.4	10	12.4	0.741

Note: Vaccinated, having received at least one recommended vaccine after HIV diagnosis; HPV, human papilloma virus; MMR, measles, mumps and rubella; Sig., significance; TDaP, tetanus diphtheria, acellular pertussis, *P*-values were obtained by the Pearson’s *χ*^2^ and Fisher’s exact test. *P*-values ≤ 0.05 are shown in bold.

### COVID-19 vaccination

COVID-19 vaccination coverage was 64.5% in the cohort. Among those vaccinated, 56.9% received the Pfizer vaccine, and 30.8% received the Johnson & Johnson (J&J) vaccine. Self-protection (65.0%) was the leading reason for vaccination, while fear of side effects (47.4%) was the primary barrier for vaccination in the unvaccinated group. COVID-19 vaccination data are presented in Table 4.

**TABLE 4:** Knowledge, history and acceptance of COVID-19 vaccination.

Question/Variable	Answer option	Total cohort (*N* = 107)	
		*n*	%
Have you ever had COVID-19?	Yes	32	29.9
	No	73	68.2
	I don’t know	2	1.9
Sex (of those who answered yes)			
Male	-	12	11.2
Female	-	20	18.7
Have you been vaccinated against COVID-19?	Yes	69	64.5
	No	38	35.5
Sex (of those who answered yes)			
Male	-	27	25.2
Female	-	42	39.3
If yes, which vaccines?	J&J	20	18.7
	Pfizer	37	34.6
	AstraZeneca	1	1.0
	Moderna	1	1.0
	Not sure	10	9.3
Received only one dose of the vaccine	J&J	12	11.2
	Pfizer	5	4.7
	AstraZeneca	0	0.0
	Moderna	0	0.0
Received two or more doses of vaccine	J&J	8	7.5
	Pfizer	32	29.9
	AstraZeneca	1	1.0
	Moderna	1	1.0
Did you experience any side effects?	Yes	36	33.6
	No	33	30.8
Sex (of those who answered yes)			
Male	-	9	8.4
Female	-	27	25.2
Which side effects were experienced?	Headache	10	9.3
	Arm pain	3	2.8
	Muscle pain	6	5.6
	Chills	4	3.7
	Fever	2	1.9
	Fatigue	9	8.4
	Drowsiness	9	8.4
	Nausea	2	1.9
	Lack of appetite	2	1.9
What influenced you to vaccinate?	Self-protection	42	39.3
	Work obligation	7	6.5
	Fear of death	3	2.8
	Social pressure	4	3.7
	Protection of others	3	2.8
What influenced you not to vaccinate?	Fear of side effects	18	16.8
	No reason	9	8.4
	No trust	6	5.6
	Religious	1	1.0
	Not aware	2	1.9

J&J, Johnson & Johnson.

### Factors associated with vaccine uptake

On univariable analysis vaccine uptake was significantly associated with female gender (*P* = 0.008), knowledge of own viral load (*P* = 0.029), having a viral load < 500 copies/mL (*P* = 0.010), knowledge of ART medication (*P* = 0.009), and receiving a recommendation from a healthcare provider (*P* = 0.001) (Table 5). In the multivariate logistic regression analysis, last viral load < 500 copies/mL and willingness to accept a recommended vaccine were excluded from the multivariate model because of quasi-separation and sparse data in certain categories, which prevented model convergence. Awareness of the influenza vaccine (adjusted OR [aOR] 4.14; 95% CI: 1.06–16.17; *P* = 0.041) and having received a recommendation from a healthcare provider (aOR 0.27; 95% CI: 0.09–0.76; *P* = 0.014) remained significantly associated with adult vaccine uptake (Table 5).

**TABLE 5:** Factors associated with vaccine uptake in a logistic regression model of a sample of people living with HIV.

Variable	Univariate (unadjusted)		*P*	Multivariate (adjusted)		*P*
	OR	95%CI		OR	95%CI	
Sex						
Male	RC	-	-	-	-	-
Female	5.00	1.38–18.06	**0.014**	3.03	0.75–12.19	0.119
Knowledge of viral load						
Yes	RC	-	-	-	-	-
No	0.36	0.14–0.92	**0.032**	0.60	0.19–1.85	0.379
Last viral load/mL						
> 500	RC	-	-	-	-	-
Undetectable	1.87	0.21–16.16	0.571	-	-	-
< 500	10.67	1.03–109.94	**0.047**	-	-	-
Knowledge of antiretroviral therapy name						
Yes	RC	-	-	-	-	-
No	0.30	0.12–0.75	**0.010**	0.54	0.18–1.57	0.256
Awareness of the influenza vaccine						
No	RC	-	-	-	-	-
Yes	7.48	2.08–26.89	**0.002**	4.14	1.06–16.17	**0.041**
Advised by doctor or nurse to get vaccinated						
Yes	RC	-	-	-	-	-
No	0.21	0.08–0.55	**0.001**	0.27	0.09–0.76	**0.014**
If recommended a vaccine, would you accept it?						
Yes	RC	-	-	-	-	-
No	0.28	0.06–1.35	0.113	-	-	-
Unsure	0.12	0.01–0.19	**0.050**	-	-	-

Note: Vaccinated, having received at least one recommended vaccine after HIV diagnosis; RC, reference category. Unadjusted OR = Odds ratios and *P*-values were derived using logistic regression models and have not been adjusted to account for other predictor variables to vaccine uptake. Multivariate OR = Odds ratios and *P*-values were derived using logistic regression models, adjusted for gender, knowledge of viral load, knowledge of antiretroviral therapy name, awareness of influenza vaccine, advised by doctor or nurse to get vaccinated. *P*-values ≤ 0.05 are shown in bold.

### Accuracy of self-reported clinical data

Among the 29 participants with both self-reported and clinically recorded viral load data, 75.9% accurately reported their viral load status (Online Appendix 1 Table 1-A1). Accurate self-reporting of CD4 counts was lowest in the < 200 cells/uL range (9.0%), and highest among those with CD4 counts > 500 cells/uL (80%) (Online Appendix 1 Table 2-A1). Variations in accuracy were also observed for vaccine-preventable conditions – for example, TB was documented in 76.6% of records but self-reported by only 56.6% of participants. Notably, influenza showed the highest agreement, with 6.7% of cases confirmed by both self-report and medical records. The data on accuracy of self-reported clinical data are shown in Online Appendix 1 (Table 3-A1).

No statistically significant association was noted between the accuracy of self-reporting of viral load or CD4 cell count and adult vaccine uptake. Among participants who accurately reported their viral load, only eight (7.5%) had received a post-HIV diagnosis vaccine. Similarly, of those who correctly reported their CD4 cell count, only three (2.8%) reported receiving a post-HIV diagnosis vaccine (Online Appendix 1 Table 4-A1).

## Discussion

People living with HIV are at heightened risk for VPDs, which can lead to severe outcomes. Despite global recommendations for routine adult immunisation in this population, real-world data on vaccine uptake remain limited, particularly in sub-Saharan Africa. This study, the first to assess uptake of recommended vaccines among PLWH in South Africa, reveals a concerningly low uptake, with only 24.3% of the cohort having received at least one adult vaccine (excluding COVID-19 vaccines) post-HIV diagnosis. By comparison, a 2016 Brazilian study reported a 60.5% vaccine uptake rate among PLWH.^26^ In our cohort, coverage was limited to only four vaccines – influenza, hepatitis B, herpes zoster (shingles), and pneumococcal vaccines, highlighting gaps in uptake of recommended vaccines according to broader HIV clinical guidelines. Influenza had the highest uptake (76.9% of vaccinated participants), likely driven by annual campaigns and availability. This finding aligns with international data presented in the study conducted in Brazil which showed that 71.8% of their cohort had received the annual influenza vaccine.^26^ In contrast, pneumococcal vaccine uptake was just 3.8%; this is similar to the 4.4% reported in a study conducted in Denmark.^27^

Our study cohort was predominantly women (67.3%), and female participants were five times more likely to have been vaccinated in univariable analysis, a finding that echoes prior research showing women engage more actively with healthcare.^28^ However, this association lost statistical significance in the final adjusted model. Most participants were over 40 years of age, and age did not significantly affect vaccine uptake. Socioeconomic challenges were apparent, with the majority of the cohort having only secondary level education (72.9%) and more than half being unemployed (54.2%). However, neither educational attainment nor employment status was significantly associated with vaccine uptake (Table 1). This supports existing evidence that HIV disproportionately affects lower socioeconomic groups,^29^ which may contribute to knowledge and barriers in accessing immunisation services.

Clinical factors proved critical predictors of vaccine uptake. Participants with a viral load < 500 copies/mL were over 10 times more likely to be vaccinated in univariable analysis. This suggests that better-controlled disease acts as an indicator for overall proactive health engagement. Prior studies reported a CD4 > 200 cells/uL was associated with increased vaccine acceptance.^16,17^ Our study found no significant association between CD4 count and vaccine uptake. This lack of correlation may reflect hesitancy among both patients and providers around vaccinating immunocompromised individuals, potentially overriding CD4 cell count status as a predictor. South African HIV management guidelines do not recommend routine CD4 cell count measurement among PLWH on ART once the CD4 cell count is above 200 cells/uL. This may result in inconsistent CD4 cell count data that is not contemporaneous.

A history of opportunistic infections was common, with TB being the most prevalent. The most common VPD was herpes zoster (shingles), followed by hepatitis B. This may explain the greater awareness of vaccines for these specific diseases. Participants who received a vaccine post-HIV diagnosis also often had children and were aware of their own childhood vaccinations, suggesting that prior exposure to or engagement with immunisation, through parenting or personal experience, may build knowledge and acceptance. Furthermore, knowledge of details of one’s HIV care was significantly associated with uptake. Those unaware of their ART medication were 70% less likely to have received vaccination, and awareness of one’s viral load also significantly increased the likelihood of uptake. This finding aligns with studies linking health literacy with proactive health decisions and underscores how empowerment through education drives better clinical outcomes.^30^ Interestingly, our analysis comparing self-reported clinical data with documented records found no statistically significant association between the accuracy of self-reporting viral load or CD4 count and adult vaccine uptake; however, this was a small subset of the total cohort. In terms of diagnosed conditions, influenza showed the highest agreement between self-report and clinical documentation.

Vaccine hesitancy persists in PLWH, often because of safety concerns, mistrust, and misinformation.^31,32,33^ Although not statistically significant, this study suggested that participants who believed vaccines were safe or prevented disease were more likely to be vaccinated. Those who did not fear adverse effects on their HIV condition also had higher acceptance. Crucially, and of great importance, a healthcare provider’s recommendation was the strongest predictor of vaccine uptake. In the final multivariate model, receiving a provider recommendation significantly increased the likelihood of being vaccinated. High willingness supports this, with 67.3% of participants indicating that they would accept a vaccine if advised by their doctor. This highlights the irreplaceable role of trusted providers in countering hesitancy and the need for clinicians to be aware of vaccination guidelines and implement them in clinical practice.

Despite only 29.9% of participants reporting having contracted COVID-19, 64.5% received a COVID-19 vaccine, a rate significantly higher than the general adult population in South Africa, as reported in a global HIV cohort study.^34^ This was likely influenced by extensive public health messaging and, potentially, vaccine mandates. Most participants cited self and family protection as their primary motivation to vaccinate. This demonstrates the power of coordinated campaigns and perceived severe threat in overcoming barriers.

### Limitations

This study has several important limitations. First, its cross-sectional design restricts the ability to establish causality between identified predictors and vaccine uptake. Second, vaccination status was primarily based on self-reporting and was not fully verified against medical records, which may have led to recall bias or overestimation of actual coverage. Third, the study was conducted at a single public-sector tertiary-level facility in an urban setting with predominantly female participants, which may limit the generalisability of findings to the broader population of PLWH in SA. Lastly, the use of convenience sampling may have introduced selection bias, as individuals who chose to participate may have been more health-conscious or more likely to be vaccinated than those who declined participation.

## Conclusion

PLWH remain disproportionately affected by VPDs, despite the availability of effective vaccines. The elevated burden of infections such as HBV, HPV, IPD, and meningococcal disease highlights a critical need for targeted immunisation strategies amongst PLWH. While global and regional guidelines exist, vaccine uptake among PLWH remains suboptimal, particularly in low- and middle-income countries like South Africa. The primary determinants of vaccine uptake identified were healthcare provider recommendation and awareness of vaccination. Key barriers include low health literacy (specifically awareness of one’s own viral load and ART status) and vaccine safety concerns. Strengthening vaccination programmes for PLWH through evidence-based policies, improved surveillance, and enhanced healthcare provider engagement is essential to reduce morbidity and mortality in this vulnerable population.
